# Supplementary material for: High-resolution simulations of chromatin folding at genomic rearrangements in malignant B cells provide mechanistic insights into proto-oncogene deregulation
Source: Genome Res. 2022 Jul;32(7):1355–66. doi: 10.1101/gr.276028.121 (PMC9341513; doi:10.1101/gr.276028.121)
Supplement: Supplemental Material [file supp_32_7_1355__DC1.html]

High-resolution simulations of chromatin folding at genomic rearrangements in malignant B cells provide mechanistic insights into proto-oncogene deregulation — High-resolution simulations of chromatin folding at genomic rearrangements in malignant B cells provide mechanistic insights into proto-oncogene deregulation — Supplemental Material 

# High-resolution simulations of chromatin folding at genomic rearrangements in malignant B cells provide mechanistic insights into proto-oncogene deregulation

## Supplemental Material

- Supplemental\_Methods.pdf
